# Long‐term outcome of transurethral laser ablation for recurrent non‐muscle invasive bladder cancer: An EORTC risk‐matched study

**DOI:** 10.1002/bco2.70052

**Published:** 2025-07-17

**Authors:** Chase Peng Yun Ng, Alexander Light, Charis Eleftheriou, Oliver Hug, Ellie Richardson, Tarra Gill‐Taylor, Altaf Shamsuddin, Hamid Abboudi, Sachin Agrawal

**Affiliations:** ^1^ Imperial Urology Imperial College Healthcare NHS Trust London UK; ^2^ Department of Surgery and Cancer Imperial College London London UK; ^3^ Department of Urology Ashford and St Peter's Hospital NHS Trust Ashford UK

**Keywords:** ablation, bladder tumour, laser, long term, non‐muscle‐invasive bladder cancer, progression, recurrence, survival

## Abstract

**Introduction:**

Elderly and comorbid patients with non‐muscle invasive bladder cancer (NMIBC) often undergo repeated transurethral resection of bladder tumour (TURBT) under general anaesthesia. Transurethral laser ablation (TULA) is an outpatient‐based alternative with lower morbidity, cost and carbon footprint, but its long‐term efficacy is not well‐established. We report the long‐term outcomes of recurrent NMIBC treated with TULA, stratified by European Organisation for Research and Treatment of Cancer (EORTC) risk groups.

**Materials and Methods:**

We conducted a single‐centre, retrospective cohort study, including all consecutive NMIBC patients treated with TULA between 2012 and 2023. The primary outcomes were recurrence‐free survival (RFS) and progression‐free survival (PFS) and secondary outcomes included cancer‐specific survival (CSS), overall survival (OS) and complications, stratified by EORTC risk groups. Data were analysed using Kaplan–Meier survival analysis and Cox regression model.

**Results:**

Three hundred and nineteen patients (1186 TULAs), with a median age of 77 and median Charleson Comorbidity Index of 7, were included. Median follow up was 4.4 years. The 5‐year RFS of intermediate‐risk and high‐risk NMIBC treated with TULA were 31.8% (95%CI:25.6–39.7%) and 29.0% (95%CI: 20.1–42.0%), respectively, with no significant difference (p:0.47). The 5‐year PFS were 86.8% (95%CI: 82.1–91.7%) and 93.1% (95%CI: 85.2–100.0%), respectively. Overall, the 10‐year OS and CSS were 50.7% (95% CI: 41.8–61.5%) and 96.1% (95%CI: 93.3–98.9%), respectively. The complication rate was 4.0%. Age was the only positive predictor of recurrence. Limitations include missing data (7.9%) and single‐centre retrospective design.

**Conclusion:**

TULA is a safe, minimally invasive treatment with long‐term efficacy for elderly and comorbid patients with recurrent NMIBC. Although recurrence rate at 5 years were high, progression rate, especially muscle invasion, was low and reassuring. Long‐term overall survival and cancer‐specific survival remained excellent.

## 1 INTRODUCTION

Bladder cancer is the most common malignancy of the urinary tract with around 10 500 new diagnoses every year.^1^ Non‐muscle invasive bladder cancer (NMIBC) accounts for approximately 75% of the cases.^2^

Fifty percent of all NMIBC are low risk.^3^ While the lifetime risk of progression and cancer‐specific mortality is relatively low, the recurrence rate can reach up to 62%, particularly in cases with large or multifocal tumours.^4^ Because of the lack of a risk‐adapted strategy, transurethral resection of bladder tumour (TURBT) remains the gold standard for all NMIBC irrespective of risk. As a result, even low‐risk patients frequently undergo repeated general anaesthesia for the procedure, exposing them to cumulative risk of surgical complications, such as bladder perforation and haematuria, as well as anaesthetic morbidity.^5^, ^6^ The high volume of TURBTs further place significant strain on health resources especially in theatre and hospital bed capacity, making bladder cancer the most expensive cancer to treat from diagnosis to death.^7^ The substantial carbon footprint aggravates effort to keep the care environmentally sustainable.^8^

There is also a need to de‐escalate and adapt management of recurrent NMIBC based on its risk. As an alternative to TURBT, office‐based laser fulguration of NMIBC is endorsed by European Association of Urology (EAU), the International Bladder Cancer Group (IBCG) and the UK Get It Right First Time (GIRFT) Bladder Cancer Commission,^3^, ^9^, ^10^ to treat low‐ and intermediate‐risk NMIBC. The aim is to treat recurrence, maximise patient satisfaction and reduce both health‐care cost and environmental impact of bladder cancer treatment.^11^, ^12^, ^13^ Our group pioneered the transurethral laser ablation of bladder tumour (TULA) procedure using dual diode lasers in 2013 to extend the scope of what is technically feasible to treat in an outpatient setting.^14^ The dual diode laser offers wavelength of 980 and 1470 nm to maximise absorption in water and haemoglobin. Blend options allow deeper laser penetration than holmium and thulium lasers, improved haemostasis, while maintaining a low risk of obturator reflex and bladder perforation. The option was first offered to high anaesthetic risk patients with low‐risk NMIBC, but with time, it was extended to comorbid patients regardless of their NMIBC risk and subsequently, during the COVID pandemic, to all patients with low‐ and intermediate‐risk NMIBC. A prospective data registry of TULA recorded no complication of post‐op bleeding in over 100 TULA procedures performed on patients with various anticoagulation.^14^

Nonetheless, data on the long‐term effectiveness of TULA for recurrent NMIBC are limited. Previous studies on outpatient‐based laser ablation were limited to holmium lasers, small sample sizes and short follow‐up durations.^15^, ^16^, ^17^, ^18^ There is also no study to date reporting the long‐term oncological outcome of TULA stratified by their NMIBC risk groups, a notable gap highlighted by Malde et al. in their systematic review.^19^ Our study adopted the 2006 European Organisation for Research and Treatment of Cancer (EORTC) and the 2021 NMIBC scoring models using the WHO 1973 classification, recommended by the European Association of Urology (EAU), to stratify the NMIBC risk at the time of first TULA for recurrence and progression, respectively.^20^, ^21^ We report the long‐term oncological outcome of recurrent NMIBC, as well as the safety and tolerability of TULA.

## 2 MATERIAL AND METHODS

This is a retrospective cohort study of all consecutive patients who underwent TULA for recurrent NMIBC at a single tertiary centre in the United Kingdom between April 2012 and July 2023. The primary outcomes were recurrence‐free survival (RFS) and progression‐free survival (PFS), stratified by estimated EORTC risk groups at the time of their first TULA. The grade and stage of the tumour is either from the histology taken at TULA or, if no biopsy was taken, the highest grade and stage of their previous TURBT specimens. The secondary outcomes were cancer‐specific survival (CSS), overall survival (OS) and complications related to TULA. We included all patients with recurrent NMIBC, irrespective of age, tumour size, tumour number, stage, grade, or prior intravesical treatments, and at least one of the following conditions:

1. Low‐risk or intermediate‐risk NMIBC.
2. High risk for general anaesthesia because of comorbidities, regardless of NMIBC risk.
3. Post‐intravesical chemotherapy instillation surveillance – bacillus Calmette–Guérin (BCG) and Mitomycin C (MMC)

Patients with incomplete data necessary to calculate the EORTC risk score and duplicates were excluded. Patients treated with radiotherapy or systemic chemotherapy and/or with less than 6 weeks of follow up were also excluded. The research is registered as a service evaluation at the Imperial College Healthcare NHS Trust. Electronic health records were reviewed to collect relevant information (Data S1).

### 2.1 Outcome definitions

The definition of recurrence and progression were adopted from the original EORTC study and recommendations from the IBCG.^21^, ^22^ Recurrence was defined as the reappearance of disease following initial TULA (previous stage/grade, high grade, T1, or CIS). Progression was a composite outcome defined as the occurrence of any of the following:

- Disease upgrade: histological increase in grade from G1 to G2/3 or G2 to G3, including CIS following the WHO 1973 classification system
- Disease upstage: presence of lamina propria invasion (increase from Ta to T1 or CIS to T1), muscle invasion (stage ≥ T2), lymph node (N+) or distant metastasis (M1)
- Bladder‐cancer related mortality

### 2.2 Procedure

All laser ablation of NMIBC was undertaken in the outpatient setting in a laser‐safe flexible cystoscopy suite using a portable dual‐diode laser, by one of four trained urology consultants. A single‐dose antibiotic (intramuscular gentamicin or oral ciprofloxacin) was given at the discretion of the surgeon prior to the procedure for symptomatic patients but not routinely needed. The procedure was performed under local anaesthetic in an aseptic manner starting with the administration of 10 ml of Instillagel®, followed by the transurethral introduction of a 16.5Fr flexible video cystoscope to survey the entire bladder mucosa for tumours using white light. Normal saline solution was used as irrigation fluid. Biopsies of tumours were usually taken prior to laser ablation, unless the surgeons decided against because of clinical reasons (for example, unlikely to impact future clinical management, risk of bleeding and unnecessary prolongation of procedure time). Where specimen is required for staging, deeper basal biopsies or resection techniques are used. Single post‐operative instillation of chemotherapy was not administered. Patients were required to wear a laser safety goggle before a dual‐diode, 1470/980 nm laser was used to ablate tumours. Complications were assessed immediately after the procedure and through routine follow up. All patients underwent a 3‐month follow‐up after laser ablation, with further cystoscopic surveillance scheduled according to the risk‐stratified EAU guideline recommendations.

### 2.3 Data source and quality assurance

Training was conducted prior to commencing data entry to ensure adherence to a standardised data entry protocol. Monitoring of the data collection was conducted by the primary researcher (PYN), and source data verification was conducted for 10% random samples when 50% and 100% of data had been collected.

### 2.4 Statistical analysis

Kaplan–Meier curves from the time of initial TULA were used to estimate the RFS, PFS, OS and CSS. Log‐rank tests were run to estimate differences in RFS and PFS between the EORTC risk groups. Multivariable Cox proportional hazard regression was used to evaluate if age, gender, Charlson Comorbidity Index (CCI) and EORTC risk groups were associated with outcomes. Multiple imputation by chain equations with 20 iterations and 1000 re‐samples was applied for missing data of 0.6% patients for CCI.

All analyses were performed using R version 4.4.1 (R Foundation for Statistical Computing, Vienna, Austria). Statistical significance was set at *p* < 0.05.

## 3 RESULTS

**FIGURE 1:**
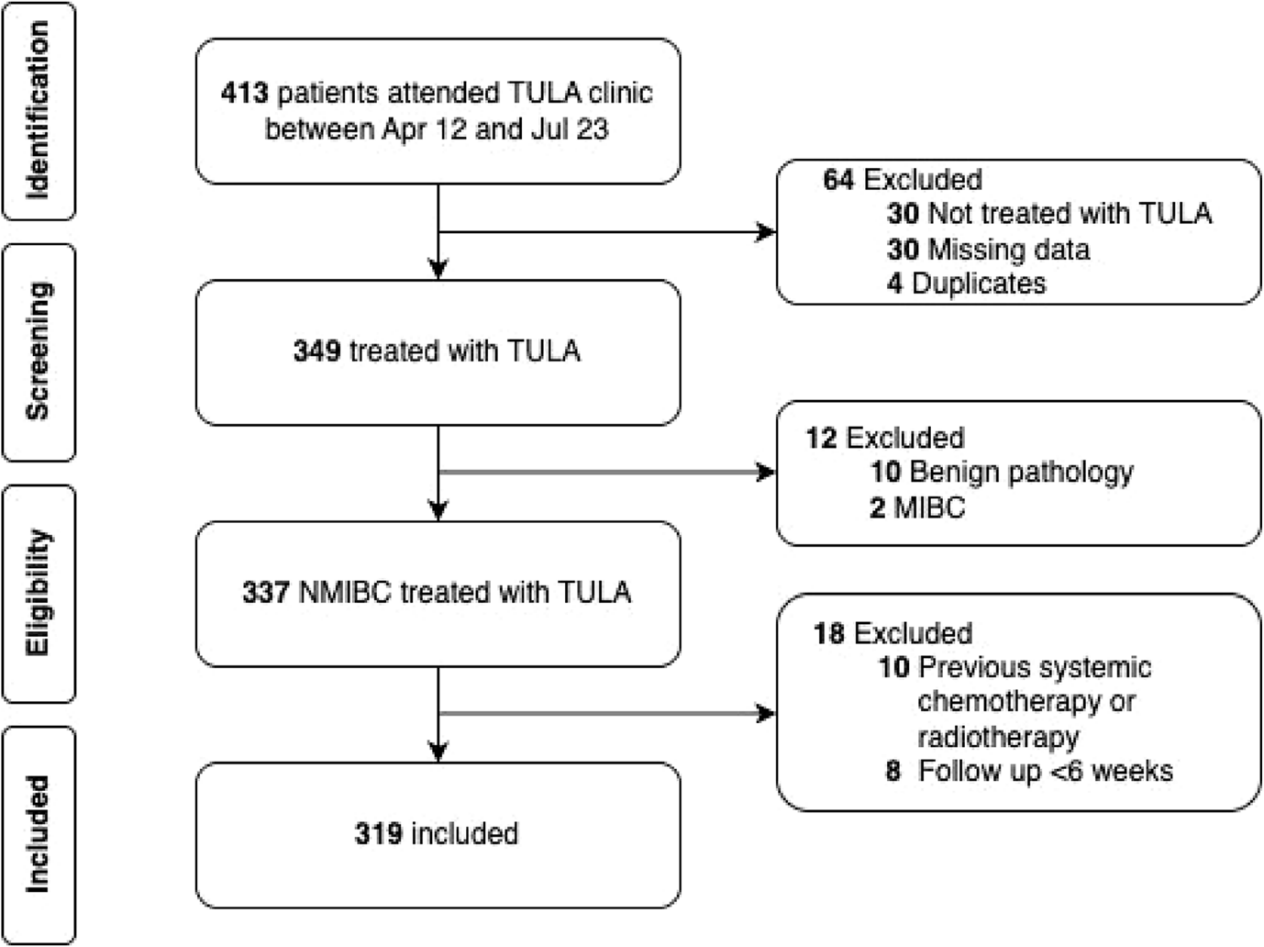
Flowchart depicting inclusion and exclusion of patients. MIBC: Muscle invasive bladder cancer.

Three hundred and nineteen patients with recurrent NMIBC, with a median age of 77 (interquartile range, IQR 68–85 years), were included (Figure 1). The median follow up was 4.4 year (IQR: 2.5–6.8 years), with 135 patients followed up for a minimum of 5 years. The median CCI was 7 (IQR 5–8). The median duration between initial NMIBC diagnosis and first TULA was 2.4 year (IQR: 1.1–4.4 years). Ninety‐nine percent (*n* = 317) of patients were stratified to intermediate‐ and high‐risk groups for recurrence, with 70% (*n* = 224) in intermediate‐risk and 29% (n = 93) in high‐risk groups, respectively (Table 1). In terms of progression, 98% (*n* = 314) of patients were stratified to intermediate‐ and high‐risk groups, with 77% (*n* = 246) in intermediate‐risk and 21% (*n* = 68) in high‐risk groups respectively. Eighty‐six (27%) patients were either receiving or have received intravesical BCG instillation and 48 (15%) patients, MMC instillation, at initial TULA. However, three patients received intravesical BCG after TULA. Overall, 1186 TULA procedures were performed. Two hundred out of 319 (62.3%) patients had biopsies at their initial TULA. The breakdown of the stage and grade of the histopathology are included as Data S1.

**TABLE 1:** Patient demographics and respective non‐muscle invasive bladder cancer (NMIBC) risk groups at initial TULA.

Characteristic	*N* = 319^a^
Age at initial TULA (years)	77 (68, 85)
Gender	
F	77 (24%)
M	242 (76%)
Charleson comorbidity index	7 (5, 8)
Unknown	2
Clinical frailty score	3 (2, 4)
Unknown	11 (3%)
ECOG performance status	
0	102 (33%)
1	115 (38%)
2	56 (18%)
3	28 (9.2%)
4	5 (1.6%)
Unknown	13
Previous Mitomycin C	48 (15%)
Unknown	2
Previous BCG	86 (27%)
Unknown	2
Highest grade	
1	60 (19%)
2	170 (53%)
3	89 (28%)
Stage	
Ta	259 (81%)
T1	60 (19%)
Largest tumour size	
< 1 cm	290 (94%)
1–2 cm	10 (3%)
2–3 cm	6 (2%)
> 3 cm	2 (1%)
Unknown	11
Number of tumour	
1–2	235 (76%)
3–5	61 (20%)
> 5	14 (5%)
Unknown	9
EORTC recurrence risk group	
2	224 (70%)
3	93 (29%)
4	2 (1%)
EORTC progression risk group	
2	246 (77%)
3	68 (21%)
4	5 (2%)

TULA, transurethral laser ablation.

^a^ Median (Q1, Q3); *N* (%).

### 3.1 Recurrence

The 1‐year and 5‐year RFS of intermediate‐risk patients were 59.9% (95%CI: 53.7–66.8%), with 88 recurrences, and 31.8% (95% CI: 25.6–39.7%), with 137 recurrences, respectively. For high‐risk patients, their 1‐year and 5‐year RFS were 54.0% (95%CI: 44.6–65.3%), with 42 recurrences, and 29.0% (95%CI: 20.1–42.0%), with 59 recurrences, respectively. No significant difference was detected (*p* = 0.47). The only two very high‐risk patients experienced recurrence within 1 year.

### 3.2 Progression

**FIGURE 2:**
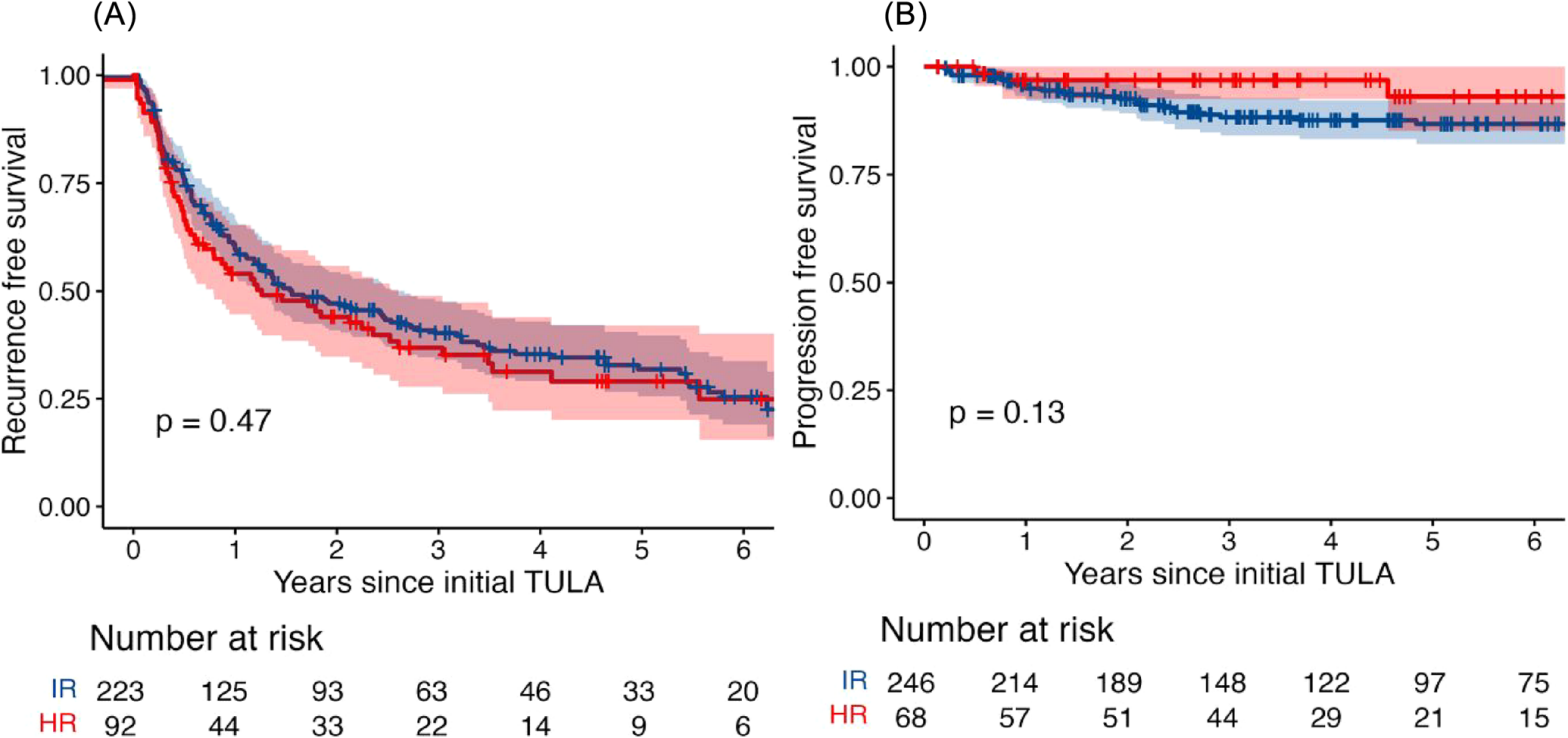
Kaplan–Meier curves for recurrence‐free and progression‐free survival stratified by EORTC risk group in NMIBC. Intermediate risk (IR): blue; high risk (HR): red; shaded areas. Shaded areas: 95% confidence intervals.

The 1‐year and 5‐year PFS of intermediate‐risk patients were 95.3% (95%CI 92.7–98.1%), with 11 progressions, and 86.8% (95%CI 82.1–91.7%) with 27 progressions, respectively (Figure 2). For high‐risk patients, their 1‐year and 5‐year PFS were 96.8% (95%CI 92.6–100.0%), with two progressions, and 93.1% (95%CI 85.2.5–100.0%), with three progressions, respectively. The log‐rank test did not demonstrate a significant difference in progression rates of these risk groups (*p*‐value = 0.13). None of five very high risk patients experienced progression.

During the follow‐up period, 31 patients (*n* = 31/319, 9.7%) experienced disease progression. Twenty six patients had their NMIBC upgraded—13 progressed from G1 to G2/3 and/or CIS and 13 progressed from G2 to G3 and/or CIS. Notably, only two patients had their disease upstaged—one from pTa to pT1 stage and one from pTa to pT2 stage—and only three patients died because of causes related to bladder cancer.

### 3.3 Mortality

**FIGURE 3:**
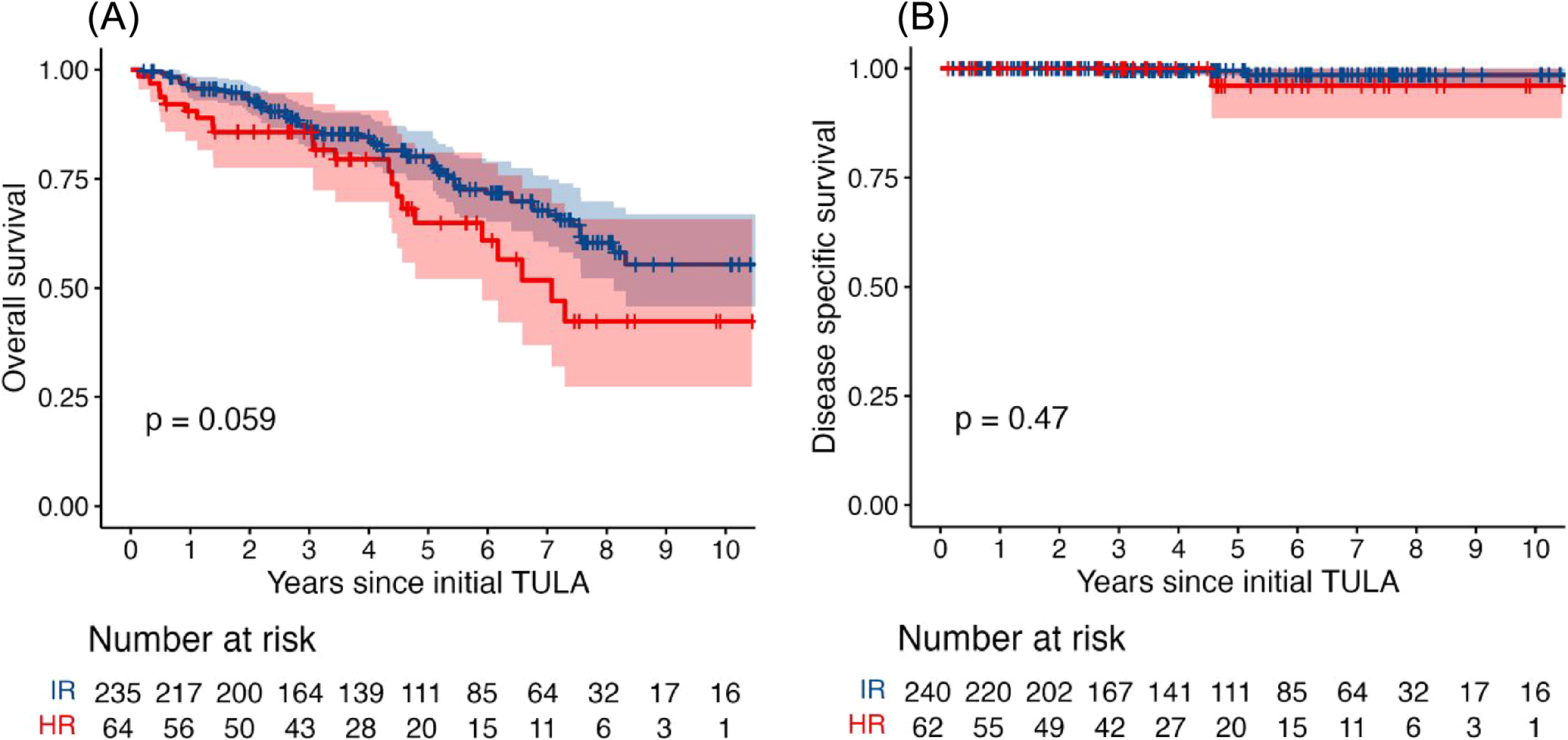
Kaplan–Meier curves for (a) overall survival and (b) cancer‐specific survival. Intermediate risk (IR): blue; high risk (HR): red; shaded areas. Shaded areas: 95% confidence intervals.

At the time of data collection, 100 patients in the cohort were deceased, of which only three were owing to bladder cancer. The median time from initial TULA to death was 3.2 years (IQR: 1.4–5.4 years). The overall 10‐year OS and CSS were 50.7% (95% CI: 41.8–61.5%) and 96.1% (95%CI: 93.3–98.9%), respectively. Intermediate‐risk patients had an estimated OS of 55.3% (95%CI 45.8–66.9%) at 10 years with median OS not reached while high‐risk patients had an estimated OS of 42.4% (95%CI 27.3–65.8%) at 10 years, with a median OS of 7.1 years. The CSS for intermediate‐risk and high‐risk patients at 10 years were estimated at 98.5% (95%CI 96.4–100.0%) and 96.0% (95%CI 88.6–100.0), respectively, with no median reached at the time of reporting (Figure 3). No significant difference in OS or CSS was detected between both risk groups.

### 3.4 Predictors

Based on the multivariable Cox proportional hazard regression models, age emerged as a positive predictor of recurrence with a hazard ratio (HR) of 1.03 (95%CI 1.01–1.04, *p* = 0.006) (Table 2). Gender, CCI and EORTC risk groups were not identified as predictors of recurrence and progression of recurrent NMIBC.

**TABLE 2:** Multivariable Cox proportional hazard regression analysis for predictors of recurrence and progression in recurrent non‐muscle invasive bladder cancer (NMIBC).

Characteristics	Recurrence			Progression		
	HR^a^	95% CI^a^	*p*‐value	HR^a^	95% CI^a^	*p*‐value
Age at initial TULA (years)	1.03	1.01, 1.04	0.006*	1.03	0.98, 1.08	0.2
Gender						
Female	—	—		—	—	
Male	1.29	0.90, 1.85	0.2	1.14	0.30, 4.32	0.8
Charleson comorbidity index	0.97	0.89, 1.04	0.4	0.96	0.72, 1.27	0.8
EORTC risk group						
2	—	—		—	—	
3	1.14	0.80, 1.62	0.5	0.92	0.15, 5.74	>0.9
4	2.72	0.66, 11.2	0.2	1.03	0.98, 1.08	0.2

^a^ HR = hazard ratio, CI = confidence interval.

* *p*‐value < 0.05.

### 3.5 Complications

Out of the 1186 procedures, 49 were excluded because of missing data. No complication was recorded in the electronic health record for 96.0% (*n* = 1092) of TULA. Around 1.5% (*n* = 17) of patients required hospital admission for haematuria; 0.4% (*n* = 5) suffer from urinary tract infection; 0.2% (*n* = 2) experienced acute urinary retention; and 0.1% (*n* = 1) abandoned procedure owing to intolerance.

## 4 DISCUSSION

Despite office‐based laser fulguration being recommended as an alternative to TURBT for low‐risk and intermediate‐risk recurrent NMIBC by EAU and NICE, there was a paucity of data on its long‐term outcomes, with the largest case series to date including only 97 highly selected patients whose NMIBC were not risk‐stratified using the holmium laser.^11^ One randomised controlled trial comparing outpatient diode laser ablation and TURBT reported recurrence at 4 months with no significant difference observed.^23^ TULA was designed using a dual diode laser to expand the scope and indications of cases that can be managed by laser fulguration under local anaesthesia. To the best of our knowledge, this is the first real‐world, risk‐matched study to report the long‐term outcome of recurrent NMIBC treated with TULA using the validated EORTC risk stratification. It is also the largest case series so far (319 patients, 1169 TULA procedures) to report on the oncological outcome, safety and tolerability of TULA.

Our patient cohort is elderly (median age of 77) and comorbid (median CCI of 7). With the haemostatic benefit of dual diode laser over holmium laser, we have a more complex case mix than the usual patient cohort for outpatient laser ablation in terms of disease burden with a quarter (25%) of patients having at least three bladder tumours and around one in 20 (6%) having bladder tumours larger than 1 cm. All patients had had at least one TURBT, and so their NMIBC were risk‐stratified based on their characteristics at initial TULA. In cases where a pragmatic approach to management was undertaken and no biopsy was taken, risk scoring was based on the most recent TURBT histopathology available. As such by default, they were at least intermediate risk. Approximately three quarters were intermediate risk and the remaining quarter was high risk. In many patients, TURBT would have been preferred but was deemed too high risk based on anaesthetic fitness.

Given this context, it is perhaps expected that the recurrence of NMIBC treated with TULA was higher than EORTC estimation at 5 years (Table 3). Interestingly, although the 5‐year progression rate for intermediate risk NMIBC was higher than EORTC estimation, that of high‐risk NMIBC was lower and comparable to EORTC estimation. Only one patient progressed to muscle invasive disease, suggesting that progression was predominantly secondary to disease upgrade. In addition, less than one in 25 patients died because of bladder cancer at 10 years. This bladder cancer‐related mortality rate is comparable to patients treated with repeat TURBT.^4^, ^20^ Approximately half of the patients including those with high‐risk NMIBC were alive by 10 years with a median OS of 7.1 years. This is reassuring, as the OS was considerably higher than the estimated 10‐year OS of someone with a median CCI of 7, which is close to 0%.^24^ In fact, our patient cohort had a median life expectancy higher than the average life expectancy of the UK population.^25^ These findings cement TULA as a safe and effective treatment with long‐term efficacy for elderly and comorbid patients with NMIBC, despite higher recurrence than EORTC estimation. Our data also suggest, while de‐escalating treatments to an outpatient local anaesthetic setting, there is a potential cancer‐specific benefit to treatment in this select groups.

**TABLE 3:** EORTC risk‐stratified 1‐ and 5‐year recurrence and progression rates of recurrent non‐muscle invasive bladder cancer (NMIBC) in patients treated with transurethral laser ablation (TULA).

TULA 1‐year recurrence rate				
EORTC RG^a^	RR^a^ (%)	SE^a^ (%)	95% CI^a^	EORTC recurrence probability^b^ (%)
Intermediate	40.1	3.3	33.2–46.3	24.0
High	46.0	5.2	34.7–56.4	48.0

TULA 5‐year recurrence rate				
EORTC RG^a^	RR^a^ (%)	SE^a^ (%)	95% CI^a^	EORTC recurrence probability^b^ (%)
Intermediate	68.2	3.6	60.3–74.4	46.0
High	71.0	5.5	68.0–79.9	62

TULA 1‐year progression rate				
EORTC RG^a^	PR^a^ (%)	SE^a^ (%)	95% CI^a^	EORTC progression probability^c^ (%)
Intermediate	4.7	1.4	1.9–7.3	0.65
High	3.2	2.2	0.0–7.4	3.8

TULA 5‐year progression rate				
EORTC RG^a^	PR^a^ (%)	SE^a^ (%)	95% CI^a^	EORTC progression probability^c^ (%)
Intermediate	13.2	2.4	8.3–17.9	3.6
High	6.9	4.2	0.0–14.8	11.0

^a^ RG = Risk Group, RR = Recurrence Rate, PR = Progression Rate, SE = Standard Error, CI = Confidence Interval.

^b^ European Organisation for Research and Treatment of Cancer (EORTC) 2006 Non‐muscle Invasive Bladder Cancer Model.

^c^ Non‐muscle Invasive Bladder Cancer 2021 Progression Scoring Model Using the WHO 1973 classification.

There is a lack of head‐to‐head comparative studies between TULA and less invasive options including active surveillance (AS) and chemoablation. However, a systematic review on AS of NMIBC reported that only three in 10 patients (32%) remained on AS after a median AS of 1.25 years with almost two in 10 (19%) experienced disease upstage or upgrade.^26^ Similarly, the BIAS cohort study of only low‐risk NMIBC showed that less than four in 10 men (37.8%) remained on AS after 18 months with six in 10 (59.6%) men requiring treatment by 3 years.^27^ These high progression and high failure rate inherently limit the durability and safety of AS in the long‐term management of NMIBC, especially in intermediate‐ and high‐risk disease. Evidence for chemoablation, using one or a combination of Mitomycin‐C (MMC), Bacillus Calmette‐Guérin (BCG), gemcitabine and epirubicin, is poor because of the heterogeneity of the regime and lack of long‐term outcome. The pooled complete response rates of chemoablation were reported to be 47.5% to 70.6% depending on the chemotherapy regime and tumour size.^19^ Two separate studies further reported the median RFS of NMIBC after 8‐weekly MCM instillation and 4‐weekly gemcitabine instillation to be 0.88 years (10.5 month) and 0.76 years (9.1 month), respectively, distinctly lower than our median RFS of 1.46 years (17.5 month).^28^, ^29^ The longest RFS reported for chemoablation, to our knowledge, was 2 years for a MMC regime (three times a week for 2 weeks). In that study, the 2‐year RFS was 36.0% (95% CI: 24–50%), whereas our 2‐year RFS was 45.9% (95%CI: 40.6–51.9%).^28^ Additionally, chemoablation strategies place a high burden of intervention and clinical visits on patients with limited morbidity data. Henceforth, within the limit of current evidence, TULA seems more effective than AS and chemoablation in terms of the oncological outcome and durability.

The safety and tolerability of TULA are also essential factors to consider. TURBT is associated with complications, such as bleeding and bladder perforation, and results in a 69% inpatient admission rate across the UK as of 2021/2022.^5^, ^30^ Based on our hospital‐based record, only 1.5% of patients treated with TULA had a hospital admission and less than 0.1% abandoned the procedure. Our finding matched complications reported by previous studies using non‐diode lasers, accepting that these may have been in a different cohort of patients (Wong et al [1.4% haematuria, 0% admission, 0% abandonment, 74 procedures], Syed et al [1.9% haematuria, 259 procedures], and Jønler et al [1.1% haematuria, 0% abandonment, 88 procedures]).^13^, ^16^, ^17^ Although our complication rate is likely an underestimation owing to loss‐to‐follow up, we did not find any worrying safety signals for TULA. A recent qualitative study reported that patients treated with TULA experienced less pain, a shorter duration of haematuria and a less negative impact on quality of life compared to patients undergone TURBT.^12^ The patients also avoided cumulative anaesthetic morbidity, such as post‐operative delirium and venous thromboembolism, especially if they were on regular anticoagulation.^31^

Lastly, it is important to identify the predictors of recurrence and progression in NMIBC treated with TULA. Our study only identified age to be a predictor of recurrence. Both EORTC NMIBC 2006 and 2021 risk models were not identified as independent variables for recurrence and progression, respectively, highlighting the gap in ways to stratify the risk of NMIBC treated with TULA in order to predict the need for retreatment and progression.

## 5 STRENGTHS AND LIMITATIONS

The strengths of this study lie in the long follow up, large sample size and EORTC risk‐stratified design in the real world setting. In the absence of a long‐term prospective comparative studies between TULA and TURBT, this study thus fills a knowledge gap that informs clinicians and patients on the long‐term efficacy of TULA.

Nonetheless, it is a retrospective study with 7.9% (*n* = 30/379) patient exclusion because of missing data. This is most likely the data missing at random owing to the loss of documentation of their initial histology (grade or stage) prior to the implementation of the electronic health record. Recurrence was not distinguished based on site (on‐site versus off‐site). Besides, because of the pragmatic real‐world approach of the study, there is a risk of underestimating disease progression rate as pathology is not available for all TULA patients. The decision to biopsy and/or for repeat TURBT because of concern of upstaging were at the discretion of the clinicians, mainly if the finding could change clinical management.

The study did not include comparative health economic and sustainability analyses. However, previous study established outpatient laser ablation to be considerably cheaper than inpatient cystodiathermy, besides releasing theatre space and time which could help cut UK national cancer wait time.^10^ Furthermore, it is also likely to be more environmentally sustainable as approximately half of TURBT carbon footprint was related to surgical equipment and anaesthesia.^8^

## 6 CONCLUSION

This study demonstrated that outpatient‐based TULA is a safe, minimally invasive treatment with long‐term efficacy for elderly and comorbid patients with recurrent NMIBC. Although recurrence rate at 5 years was high, progression rate, especially to muscle invasion, was low and reassuring. Long‐term overall survival and cancer‐specific survival remained excellent. A head‐to‐head comparative study of TURBT, TULA, chemoablation and active surveillance is needed to better inform a risk‐adapted strategy to manage NMIBC.

## AUTHOR CONTRIBUTIONS

HA, SA and PN conceived and designed the study. ER, OH, CE and TG extracted the data. PYN verified the data with discrepancy discussed with HA. AL and PN analysed, visualised and interpreted the data. PN drafted the manuscript. AL, HA and SA reviewed and edited drafts of the manuscript. All authors read the manuscript, provided feedback and approved the final version.

## CONFLICT OF INTEREST STATEMENT

SA has received funding for education and training activity from Biolitec and Promed UK.

## Supporting information


**Data S1.** Supporting Information
