# Supplementary material for: Long‐term outcome of transurethral laser ablation for recurrent non‐muscle invasive bladder cancer: An EORTC risk‐matched study
Source: BJUI Compass. 2025 Jul 17;6(7):e70052. doi: 10.1002/bco2.70052 (PMC12270553; doi:10.1002/bco2.70052)
Supplement: Supplementary file 1 — Data S1. Supporting Information [file BCO2-6-e70052-s001.docx]

**Supplementary Data**

1. **Breakdown of TULA Histopathology**

| TULA Pathology | n = 200 |
| --- | --- |
| Highest Grade |  |
| 1 | 33 (16.5%) |
| 2 | 77 (38.5%) |
| 3 | 23 (11.5%) |
| CIS | 2 (1%) |
| Benign | 65 (32.5%) |
| Stage |  |
| Ta | 121 (60.5%) |
| T1 | 14 (7.0%) |
| Not applicable | 65 (32.5%) |
